# Suppression of protein kinase C theta contributes to enhanced myogenesis *In vitro* via IRS1 and ERK1/2 phosphorylation

**DOI:** 10.1186/1471-2121-14-39

**Published:** 2013-09-21

**Authors:** Joseph S Marino, Terry D Hinds, Rachael A Potter, Eric Ondrus, Jeremy L Onion, Abigail Dowling, Thomas J McLoughlin, Edwin R Sanchez, Jennifer W Hill

**Affiliations:** 1 Center for Diabetes and Endocrine Research, Department of Physiology and Pharmacology, University of Toledo College of Medicine, Toledo, OH 43614, USA; 2 Department of Kinesiology, University of Toledo, Toledo, OH 43606, USA; 3 Department of Kinesiology, Laboratory of Systems Physiology, University of North Carolina at Charlotte, Charlotte, NC 28223, USA

**Keywords:** Protein kinase C, Myoblast differentiation, Myoblast fusion, Insulin receptor substrate

## Abstract

**Background:**

Differentiation and fusion of skeletal muscle myoblasts into multi-nucleated myotubes is required for neonatal development and regeneration in adult skeletal muscle. Herein, we report novel findings that protein kinase C theta (PKCθ) regulates myoblast differentiation via phosphorylation of insulin receptor substrate-1 and ERK1/2.

**Results:**

In this study, PKCθ knockdown (PKCθ^shRNA^) myotubes had reduced inhibitory insulin receptor substrate-1 ser1095 phosphorylation, enhanced myoblast differentiation and cell fusion, and increased rates of protein synthesis as determined by [^3^H] phenylalanine incorporation. Phosphorylation of insulin receptor substrate-1 ser632/635 and extracellular signal-regulated kinase1/2 (ERK1/2) was increased in PKCθ^shRNA^ cells, with no change in ERK5 phosphorylation, highlighting a PKCθ-regulated myogenic pathway. Inhibition of PI3-kinase prevented cell differentiation and fusion in control cells, which was attenuated in PKCθ^shRNA^ cells. Thus, with reduced PKCθ, differentiation and fusion occur in the absence of PI3-kinase activity. Inhibition of the ERK kinase, MEK1/2, impaired differentiation and cell fusion in control cells. Differentiation was preserved in PKCθ^shRNA^ cells treated with a MEK1/2 inhibitor, although cell fusion was blunted, indicating PKCθ regulates differentiation via IRS1 and ERK1/2, and this occurs independently of MEK1/2 activation.

**Conclusion:**

Cellular signaling regulating the myogenic program and protein synthesis are complex and intertwined. These studies suggest that PKCθ regulates myogenic and protein synthetic signaling via the modulation of IRS1and ERK1/2 phosphorylation. Myotubes lacking PKCθ had increased rates of protein synthesis and enhanced myotube development despite reduced activation of the canonical anabolic-signaling pathway. Further investigation of PKCθ regulated signaling may reveal important interactions regulating skeletal muscle health in an insulin resistant state.

## Background

Skeletal muscle development and the regeneration of adult muscle tissue requires the completion of myogenesis: activation, proliferation, differentiation, and fusion of muscle specific stem cells, known as satellite cells [1]. Myogenesis is highly regulated by cellular, molecular, and particularly hormonal signals that orchestrate cell mobility, cell contact, hormone sensitivity and the expression of muscle regulatory factors (i.e. MyoD, Myf5, myogenin, and MRF5) [2,3].

Hormone signaling is critical in the regulation of skeletal muscle mass. Mitogenic signals from insulin and insulin-like growth factor (IGF-1) converge on the insulin receptor substrate (IRS) to regulate cell metabolism, protein synthesis, cell survival, and cell growth by activating phosphoinositide 3-kinase (PI3-kinase)/protein kinase B (PKB or AKT) and extracellular signal-regulated kinase (ERK) signaling pathways [4-9]. However, the kinases and the mechanisms that regulate signal transduction through these cascades, as well as the result on myogenesis, are not completely characterized. Specifically, PI3-kinase is a primary regulator of anabolic and catabolic responses that contribute to the maintenance of skeletal muscle mass, and is activated by IRS1 [10,11]. Importantly, the theta isoform of the protein kinase C family (PKCθ) phospho-inhibits insulin receptor substrate-1 (IRS1) on ser1101 (homologous to ser1095 mouse numbering), suppressing downstream activation of AKT [12], a target of PI3-kinase and mediator of anabolic and catabolic signaling [10,11]. PKCθ also regulates skeletal muscle regeneration *in vivo*[13] and myogenesis *in vitro*[14-16], albeit through mechanisms that are not completely understood. Therefore, further investigation into the cellular signaling dynamics regulated by PKCθ will advance our understanding of the cellular and molecular regulation of the myogenic program.

PKC molecules are intracellular serine/threonine kinases expressed by a variety of cell types involved in diverse functions depending on their structure. PKC molecules are classified as either 1) conventional, containing Ca^2+^ and diacylglycerol/phorbol binding domains, 2) novel, missing the Ca^2+^ binding domain and 3) atypical, lacking the Ca^2+^ and diacylglycerol binding domains [17]. PKCθ is a member of the novel family of PKC molecules and is predominantly expressed in hematopoietic [18] and skeletal muscle cells [19].

In skeletal muscle, PKCθ regulates, insulin sensitivity [20-22], muscle cell proliferation and differentiation [14,16,23], skeletal muscle regeneration [13], and expression of acetylcholine receptors in the neuromuscular junction [24-26]. Nonetheless, the contribution of PKCθ to myogenesis is controversial. Studies using human [23] and chick [15] primary muscle cells showed that PKCθ expression decreases throughout differentiation, a time associated with increased muscle creatine kinase [15] and desmin [14] protein levels, both of which support differentiation and myotube formation. PKCθ was not detected in mouse embryonic myoblasts, which were resistant to the inhibitory effects of phorbol esters and transforming growth factor beta (TGF-β) [27,28] on myotube formation [29]. Genetic forced expression of PKCθ in mouse embryonic myoblasts prevented myotube formation in the presence of TGFβ and phorbol ester [29]. Moreover, mice with dystrophic muscle have improved skeletal muscle regeneration when PKCθ is globally absent [13]. Taken together, these studies support that PKCθ is a negative regulator of myogenesis and skeletal muscle regeneration. Alternatively, primary muscle cell cultures derived from global PKCθ knockout mice and muscle-specific PKCθ kinase-dead mice have demonstrated a requirement for PKCθ in myogenesis and regeneration [16]. Lastly, in C_2_C_12_ muscle cells, PKCθ expression remained constant and overexpression of PKCθ did not impair differentiation [30].

The overall objective of this study was to investigate how PKCθ regulates cell signaling events that contribute to the advancement of the myogenic program. We hypothesized that PKCθ negatively regulates the myogenic program via IRS1. To test this hypothesis we used a short hairpin-RNA (shRNA) to specifically knockdown PKCθ expression in C_2_C_12_ cells (PKCθ^shRNA^), an established cell line for investigating the myogenic program [8,30-32]. We then investigated how reduced PKCθ affected signaling through the classical insulin signaling pathway in addition to the affect on differentiation and fusion of muscle myoblasts. Our data reveal a PKCθ-regulated myogenic pathway involving serine phosphorylation of IRS1 and phosphorylation of ERK1/2 in the control of myoblast differentiation that enhances our understanding of how PKCθ contributes to myogenic signaling.

## Results and discussion

### Knockdown of PKCθ in C_2_C_12_ cells

**Figure 1:**
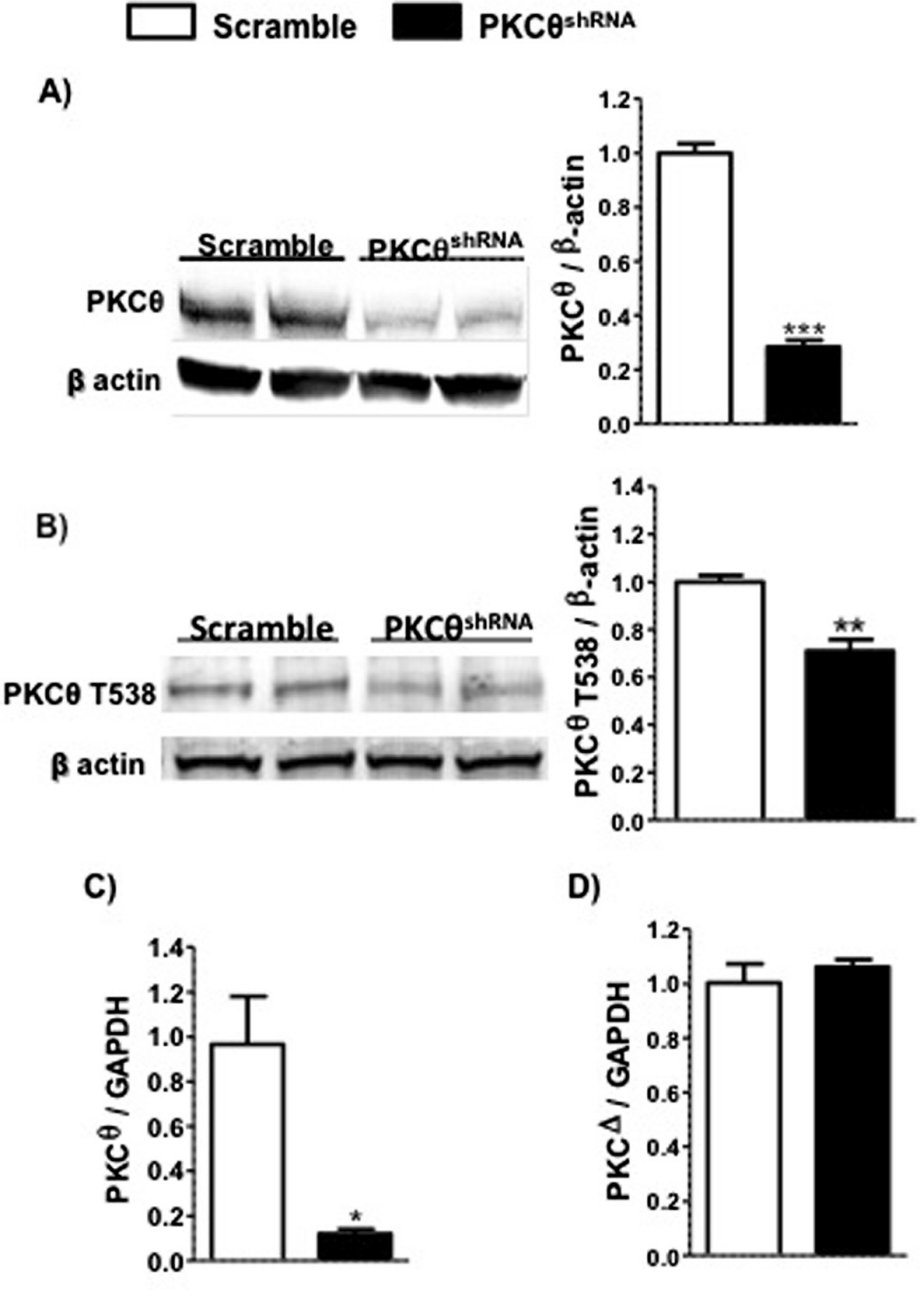
**Targeted knockdown of PKCθ. A**. Western blot for PKCθ in scramble and PKCθ^shRNA^ cells normalized to β-actin. ***; P<0.0001. **B)** Western blot for PKCθ T538 in scramble and PKCθshRNA cells normalized to β-actin. **; P=0.0064. **C)** PKCθ gene expression in scramble and PKCθ^shRNA^ cells. *; P=0.017. **D)** PKCΔ gene expression in scramble and PKCθ^shRNA^ cells. Data ± SEM. n=3 for all experiments.

To investigate the mechanism by which PKCθ regulates muscle cell differentiation and fusion, a stable PKCθ knockdown (PKCθ^shRNA^) cell line using C_2_C_12_ mouse muscle cells (myoblasts) was generated by transfecting with a PKCθ shRNA. Transfection reduced PKCθ protein and gene expression by approximately 80% compared to cells transfected with scramble oligonucleotides (scramble) (Figures 1A,C). Additionally, phosphorylation of PKCθ was significantly reduced in PKCθ^shRNA^ myoblasts (Figure 1B). Gene expression of PKC delta (PKCΔ), also a member of the novel family of PKC molecules, was not different between PKCθ^shRNA^ and scramble myoblasts (Figure 1D), indicating specificity of the shRNA.

### PKCθ is a negative regulator of myogenesis in C_2_C_12_ muscle cells

**Figure 2:**
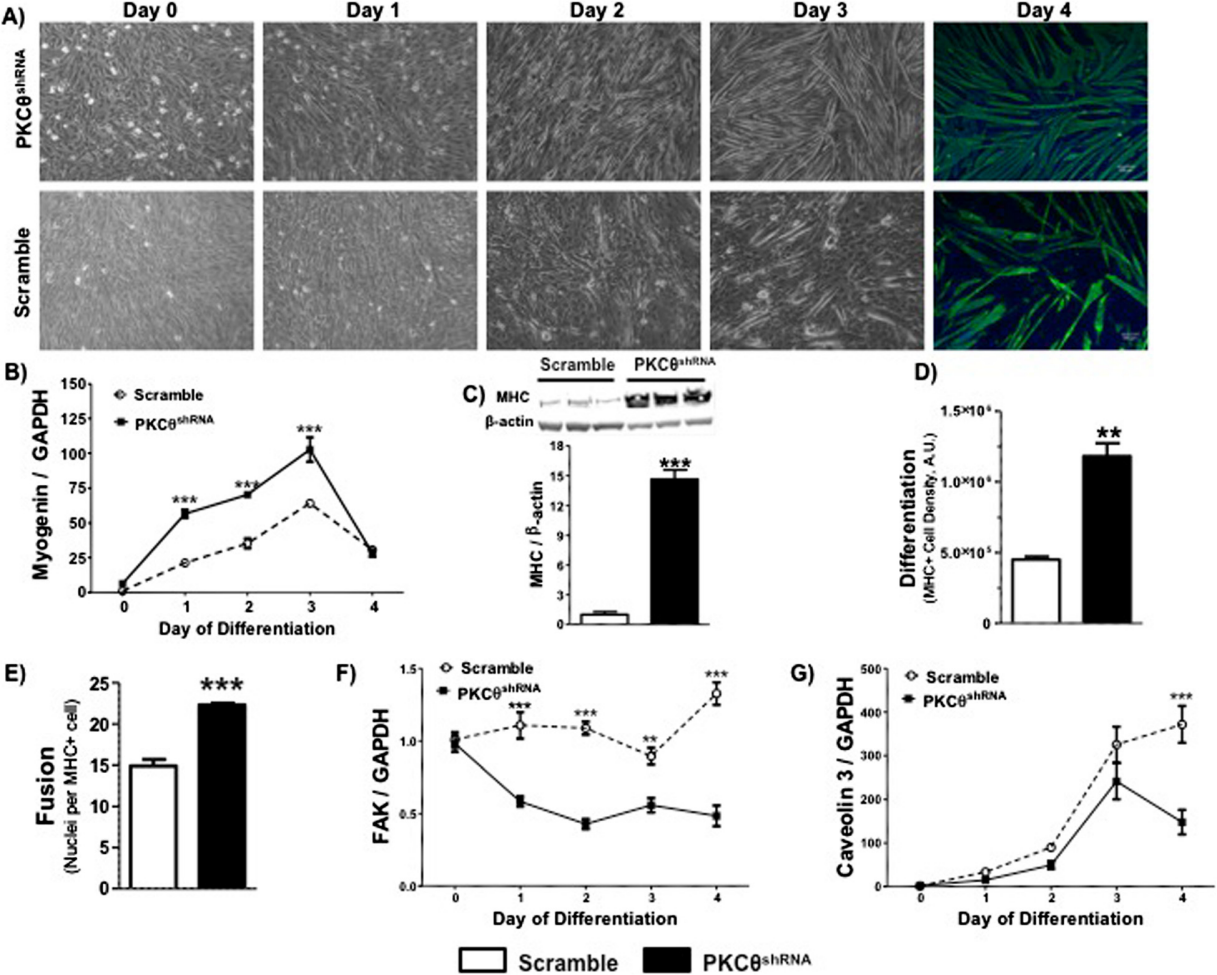
**PKCθ regulates the myogenic program. A)** Light microscopy images taken at 10X of scramble and PKCθ^shRNA^ cells from Day 0 through Day 4 of differentiation. On Day 4 cells were stained for MHC expression (green) and nuclei counterstained with DAPI (Blue). **B)** Time course of myogenin gene expression in differentiating scramble and PKCθ^shRNA^ cells. ***; P<0.0001 between scramble and PKCθ^shRNA^ cells at indicated time points. **C)** MHC protein expression in scramble and PKCθ^shRNA^ day 4 myotubes normalized to β-actin. ***; P<0.0002. **D)** Quantification of the density of MHC+ cells. **; P=0.0015. **E)** Number of nuclei per MHC+ cell. ***; P=0.0008. **F)** Time course of Focal adhesion kinase (FAK) gene expression in differentiating scramble and PKCθ^shRNA^ cells. **; P<0.01 and ***; P<0.001 between scramble and PKCθ^shRNA^ at indicated time points. **G)** Time course of caveolin 3 gene expression in differentiating scramble and PKCθ^shRNA^ cells. ***; P<0.001 at indicated time point. Data ± SEM. n=3 for all experiments.

To determine how the loss of PKCθ affects differentiation and fusion of myoblasts, PKCθ^shRNA^ and scramble cells were exposed to differentiation media for 4 days. On day 2, PKCθ^shRNA^ cells formed a greater number of tube-like structures compared to scramble cells (Figure 2A, Day 2 pictures). This is in agreement with increased myogenin transcript levels from day 1 through day 3 of differentiation in PKCθ^shRNA^ cells (Figure 2B). On the fourth day, cells were stained for myosin heavy chain (MHC) to identify differentiated cells and counterstained with DAPI to identify nuclei (Figure 2A, Day 4). MHC protein expression via western blot (Figure 2C) and immuno staining (Figure 2D) were markedly increased, approximately 15 fold and 2.5 fold respectively, in PKCθ^shRNA^ compared to scramble cultures. In addition, the number of nuclei per MHC+ cell, an indication of cell fusion, was 20% greater in PKCθ^shRNA^ cultures (Figure 2E), indicating PKCθ is a myogenic suppressor of C_2_C_12_ myoblast differentiation and fusion.

Focal adhesion kinase (FAK) and caveolin 3 are necessary for myoblast fusion and *in vivo* regeneration [33,34]. Here, the gene expression of FAK (Figure 2F) and caveolin 3 (Figure 2G) were analyzed through 4 days of differentiation. Interestingly, mRNA levels of FAK remained lower in PKCθ^shRNA^ compared to scramble cells from day 1 through day 4 of differentiation (Figure 2F). Caveolin 3 mRNA levels remained similar between cell types from day 1 through day 3 of differentiation. At day 4 of differentiation, caveolin 3 levels dropped in PKCθ^shRNA^ myotubes while increasing slightly in the scramble culture resulting in a significant difference (Figure 2G).

A decrease in FAK protein expression was reported following 96 hours of differentiation [34], which supports our results. Furthermore, FAK regulates the expression of caveolin 3 [34]. Therefore, reduced expression of caveolin 3 reported here might be the result of down-regulated FAK. The lower expression levels of both FAK and caveolin 3 in our PKCθ^shRNA^ cells following 4 days of differentiation support the acceleration of the fusion process compared to scramble cultures. It is possible that FAK expression peaks in PKCθ^shRNA^ cells at an earlier time point than analyzed here, propagating accelerated myotube development. Alternatively, muscle cells derived from global PKCθ knockout mice (PKCθ−/−) have impaired myogenic properties *in vitro* associated with reduced FAK and caveolin 3 [16]. Importantly, expression levels of FAK and caveolin 3 were analyzed after 2 days in differentiation conditions [16], while cells in this study were differentiated for 4 days prior to analysis. Indeed, primary cultures derived from PKCθ−/− display impaired fusion *in vitro*[16], which is in contrast to our data here, derived from C_2_C_12_ cells in which shRNA was used to knockdown PKCθ expression. Although differences between a primary culture and cell line may contribute to the desperate findings, the *in vivo* milieu is complex and dynamic, and cellular interactions between inflammatory and skeletal muscle cells, two sources of PKCθ [18,19], may promote changes in cellular function that alter *ex vivo* cellular dynamics. Inflammatory cells play an integral role in regulating skeletal muscle size [35].

Primary mouse muscle cells isolated from skeletal muscle PKCθ kinase-dead mice also have impaired myogenic properties and regeneration *in vivo*[16], contrary to results presented in this study. Importantly, PKCθ translocates to the nucleus in cultured human muscle satellite cells [14] and other cell types where it directly associates with chromatin [36]. Also, in T-cells, PKCθ directly binds cytosolic proteins to regulate activity [37]. Together, these findings demonstrate that PKCθ has functions beyond its kinase activity including protein-protein interactions and protein-DNA interactions that remain to be completely explored in skeletal muscle. These functions of PKCθ may explain the contradictory results obtained with our model compared to other models, which rely on substrate binding and availability [16]. Indeed, mice with muscular dystrophy and the additional global null mutation for PKCθ, have enhanced skeletal muscle regeneration [13], suggesting a negative role for PKCθ in the regulation of myogenesis. Further work exploring the cellular and molecular interactions of skeletal muscle PKCθ across multiple models is warranted to more completely understand its myogenic regulatory role.

### Lack of PKCθ enhances protein synthesis apart from classical IRS1 signaling

**Figure 3:**
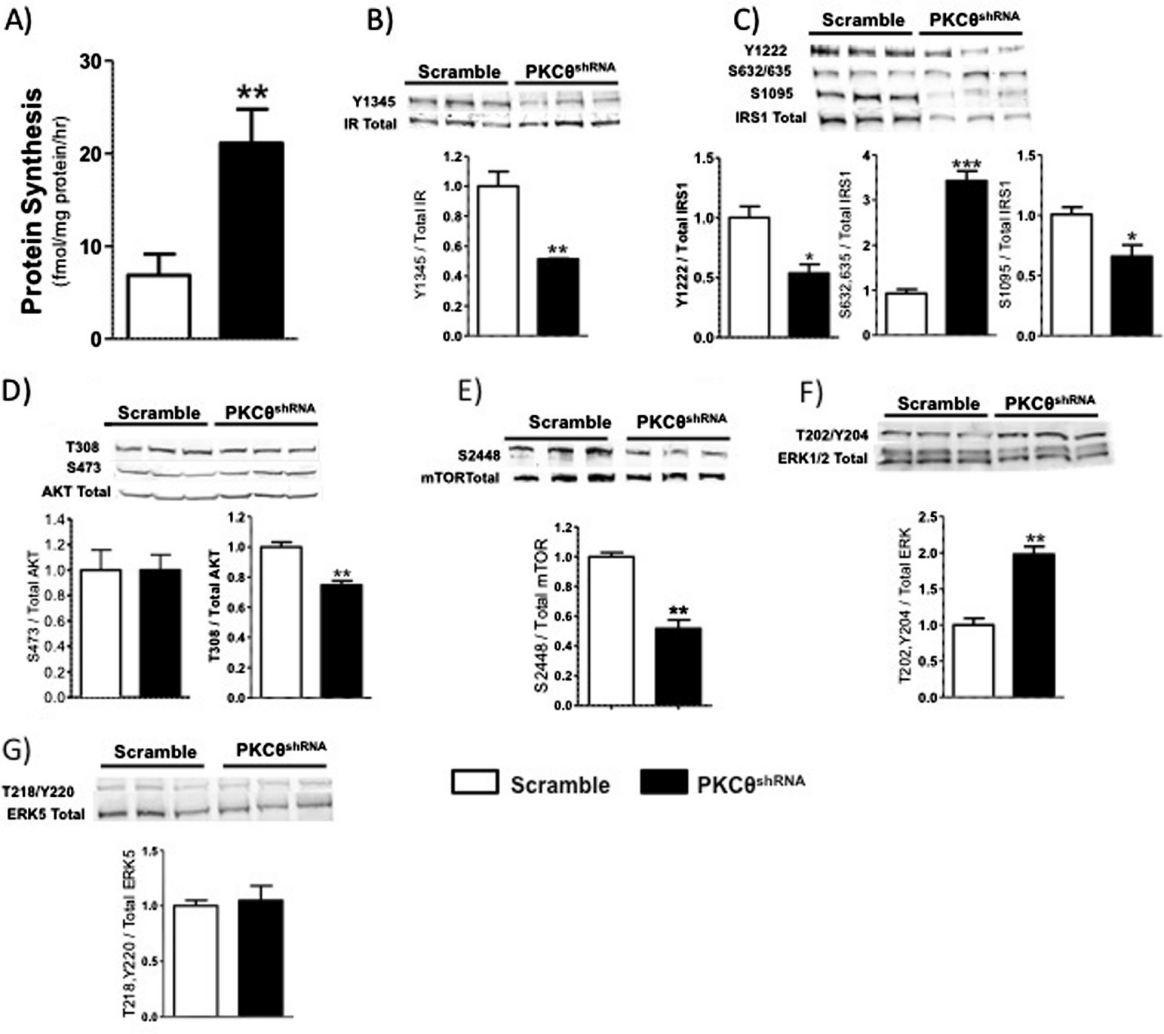
**Lack of PKCθ enhances protein synthesis apart from classical IRS1 signaling. A)** Rates of protein synthesis determined after 4 days in differentiation media. **; P=0.0082. **B)** IR tyrosine phosphorylation normalized to total IR protein. **; P<0.0077. **C)** IRS1 tyrosine and serine phosphorylation normalized to total IRS1 protein. *; P<0.05. ***; P=0.0005. **D)** AKT phosphorylation normalized to total AKT protein. **; P=0.0042. **E)** mTOR phosphorylation normalized to total mTOR. **; P=0.0017 **F)** ERK1/2 phosphorylation normalized to total ERK1/2 protein. **; P=0.0023. **G)** ERK5 phosphorylation normalized to total ERK5 protein. Data ± SEM. n=3 for all experiments.

Our data indicates that PKCθ negatively regulates the differentiation and fusion of myoblasts. Because PKCθ inhibits IRS1 through serine phosphorylation and this results in the downstream suppression of AKT [12], we tested the hypothesis that PKCθ regulates myoblast differentiation and fusion through altered IRS1 signaling. IRS1 signal transduction regulates cell growth and protein synthesis through PI3-kinase/AKT activation and the MAPK cascade involving MEK1/2/ERK signaling [5,6,8,9,38,39]. IRS1 serine phosphorylation of specific residues inhibits downstream signaling by preventing IRS1 tyrosine phosphorylation [4,12,22,40]. Specifically, phosphorylation of serine1095 (referenced as 1101 human numbering) by PKCθ impairs insulin signaling [12]. In support of our hypothesis, PKCθ^shRNA^ cells had elevated rates of protein synthesis determined by [^3^H] phenylalanine incorporation (Figure 3A), accompanied by reduced IRS1 serine1095 phosphorylation following 4 days of differentiation (Figure 3C). However, myogenic events are likely independent of insulin receptor (IR) signaling because its tyrosine phosphorylation was lower in PKCθ^shRNA^ cells (Figure 3B) despite increased differentiation, fusion, and protein synthesis (Figures 2 and 3A). Furthermore, IRS1 phosphorylation at tyrosine 1222 was reduced in PKCθ^shRNA^ myotubes (Figure 3C). Moreover, phosphorylation of AKT, a kinase activated in response to IRS1/PI3-kinase signaling [4,41], was not different between cell types at serine 473, however was reduced in PKCθ^shRNA^ myotubes at threonine 308 (Figure 3D). Lastly, phosphorylation of mammalian target of rapamycin (mTOR) at serine 2448, a downstream target of AKT, was also reduced in PKCθ^shRNA^ day 4 myotubes (Figure 3E). Collectively, our protein synthesis and immunoblot data suggests involvement of a mechanism other than the canonical IRS1/PI3-kinase/AKT signaling pathway in promoting differentiation, fusion and protein synthesis in PKCθ^shRNA^ cells.

MAPKs participate in the regulation of a plethora of cellular functions, including the proliferation and differentiation of muscle cells and the modulation of IRS1 signaling. Specifically, ERK1/2 expression increases during differentiation of C_2_C_12_ cells [5,42] and permits the expression of myosin heavy chain [32]. Furthermore, ERK5 regulates myogenesis in a pathway independent of that, which activates MyoD, and MEF2 regulated genes [8]. Moreover, MEK1/2 is a positive regulator of the muscle specific transcription factor MyoD whose expression is required for the initiation of myoblast differentiation [5]. ERK also reciprocally signals to IRS1 [4,40,43,44]. In 3T3-L1 cells, IRS1 serine 636/639 phosphorylation causes IRS1 degradation [43] which is dependent on MEK1/2-induced ERK activation in human skeletal muscle cells [45]. Finally, in myeloma cells, ERK is phosphorylated through an IRS1-dependent mechanism [44]. In this study, total IRS1 protein levels were markedly reduced in PKCθ^shRNA^ cells together with increased phosphorylation of serine 632/635 (mouse numbering homologous to human 636/639) (Figure 3C) in day 4 myotubes, suggesting ERK-dependent signaling. As anticipated, ERK1/2 phosphorylation was increased in PKCθ^shRNA^ cells (Figure 3F). While ERK5 has been demonstrated to also regulate fusion of C_2_C_12_ muscle cells [8], a difference in ERK5 phosphorylation between PKCθ^shRNA^ and scramble cultures was not detected (Figure 3G). While phosphorylation sites on ERK5 other than those analyzed here contribute to cell growth an survival in other cell types, these sites have been shown regulate mitotic activity [46,47] rather than terminal differentiation.

Interestingly, mTOR has been identified as a substrate for ERK [48], and mTOR is required for the fusion of differentiated skeletal muscle cells [31]. Skeletal muscle overexpression of Rheb increased mTOR mediated kinase events resulting in increased skeletal muscle size and protein translation independent of PI3-kinase and PKB (AKT) [49]. Here, mTOR phosphorylation was reduced in PKCθ^shRNA^ day 4 myotubes suggesting that mTOR is not a prime regulator of protein synthesis and myotube development in cells lacking PKCθ at the time point analyzed.

Our data together with prior reports [43,45] support that lack of PKCθ in C_2_C_12_ myotubes promotes ERK1/2 mediated phosphorylation of IRS1 at serine 632/635. While this mechanism corroborates our finding of reduced total IRS1 protein (Figure 3C), further work is required to determine the mechanism by which these signaling events lead to enhanced protein synthesis. Nonetheless, these data show a novel pathway by which protein synthesis is increased despite reduced insulin receptor and AKT phosphorylation.

### PKCθ regulates IRS1 and ERK-mediated differentiation

**Figure 4:**
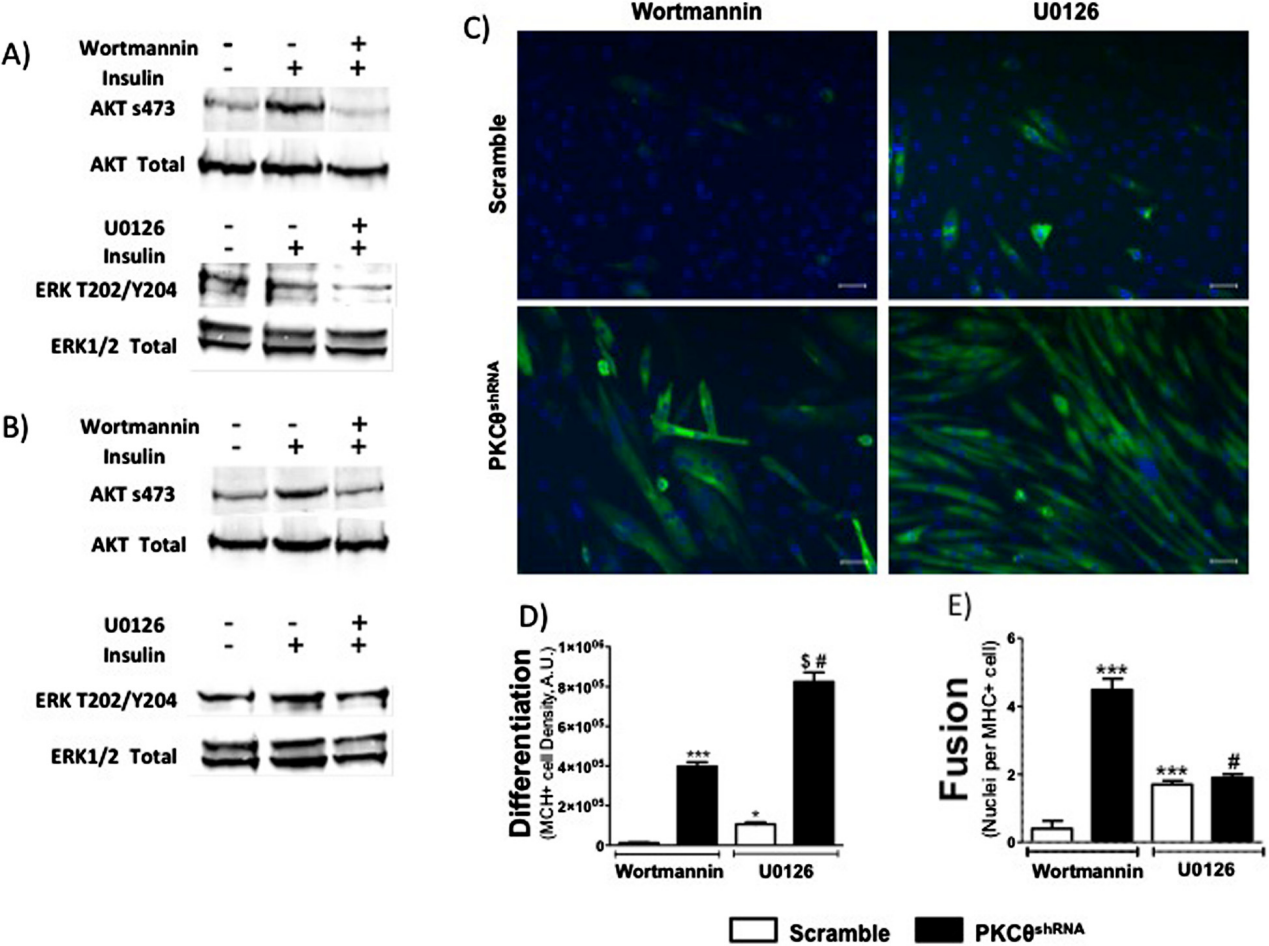
**PKCθ regulates IRS1 and ERK-mediated differentiation. A)** Western blot analysis of AKT s473 and ERK T202/Y204 in scramble and **B)** PKCθ^shRNA^cells treated with insulin and with or without wortmannin and U0126.**C)** 10x immunoflourescence images of scramble and PKCθ^shRNA^ cells treated with wortmannin and U0126 (Green; MHC and Blue; nuclei). **D)** Quantification of the density of MHC+ cells. *; P<0.05 and ***; P<0.0001 compared to wortmannin treated scramble cells. $; P<0.0001 compared to U0126 treated scramble cells. #; P<0.0001 compared to wortmannin treated PKCθ^shRNA^ cells. **E)** Number of nuclei per MHC+ cell. ***; P<0.0001 compared to wortmannin treated scramble cells. #; P<0.0001 compared to wortmannin treated PKCθ^shRNA^ cells. Data ± SEM. n=3 for all experiments.

The purpose of these studies was to determine which kinases downstream of IRS1 mediate myoblast differentiation and fusion in PKCθ^shRNA^ cells. Scramble and PKCθ^shRNA^ cells were treated with the PI3-kinase inhibitor wortmannin to attenuate PI3-kinase/AKT activation (Figure 4A) or the MEK1/2 inhibitor U0126 to inhibit ERK activity (Figure 4B). Wortmannin completely blocked the expression of MHC and subsequent cell fusion in scramble cells (Figures 4B,C,D), consistent with prior reports [50]. U0126 drastically reduced MHC expression and fusion in scramble cells compared to untreated cultures (Figure 2A compared to Figure 4A). However, expression of MHC was greater in U0126 compared to wortmannin treated scramble cells, indicating a greater degree of differentiation (Figures 4C,D). While the number of nuclei per MHC+ cell was statistically greater in U0126 compared to wortmannin treated scramble cultures, fewer than 2 nuclei per MHC+ cell indicates markedly impaired fusion (Figure 4E).

Compared to wortmannin treated scramble cells, PKCθ^shRNA^ cells had increased differentiation and maintained the ability to fuse despite the presence of the PI3-kinase inhibitor (Figures 4B,C,D). Moreover, PKCθ^shRNA^ myotubes maintained higher rates of protein synthesis when treated with wortmannin compared to scramble cultures. Specifically, in agreement with figure 3A, protein synthesis was approximately 2-fold higher in PKCθ^shRNA^ compared to scramble day 4 myotubes exposed to vehicle (4.8 vs. 9.2 fmol/mg protein/hr; P <0.05). In response to wortmannin, PKCθ^shRNA^ protein synthesis rates remained 35% higher in PKCθ^shRNA^ compared to scramble myotubes (3.17 vs. 4.90 fmol/mg protein/hr; P <0.05). Thus, PKCθ^shRNA^ cells are able to complete the myogenic program independent of PI3-kinase signaling. These results support our protein expression data (Figure 3) in which reduced IR and AKT phosphorylation were found in PKCθ^shRNA^ compared to scramble day 4 myotubes. Importantly, wortmannin treatment of PKCθ^shRNA^ reduced differentiation to levels comparable to untreated scramble cultures (Figure 2A and Figure 4D). Therefore, while lack or PKCθ in C_2_C_12_ myotubes is permissive for differentiation despite PI3-kinase inhibition, PI3-kinase signaling may be necessary to manifest the enhanced and accelerated myotube development observed in untreated cultures.

PKCθ^shRNA^ cells treated with U0126 had markedly increased density of MHC+ cells (Figures 4C,D). Cell fusion, on the other hand, as determined by nuclei per MHC+ cell, was not different between PKCθ^shRNA^ and scramble cells in the presence of the MEK inhibitor (Figures 4C,E). There was also no difference in protein synthesis rates between PKCθ^shRNA^ and scramble myotubes treated with U0126 (4.2 vs. 5.0 fmol/mg protein/hr). shRNA-mediate reduction of PKCθ protected muscle cell differentiation in the presence of both PI3-kinase and MEK1/2 inhibition, but cell fusion was protected only in the presence of PI3-kinase inhibition. Take together, these data show that MEK1/2 signaling is required for cell fusion independently of differentiation and the expression of PKCθ. Furthermore, our data suggests a PKCθ-specific myogenic regulatory pathway involving IRS1 and ERK1/2 phosphorylation events in the regulation of muscle cell differentiation.

## Conclusions

The objective of this study was to investigate the contribution of skeletal muscle cell PKCθ to signaling events that regulate protein synthesis and myogenesis. Taken together, our data supports a model in which PKCθ regulates IRS1 and ERK1/2 signaling that controls myoblast differentiation and protein synthesis. Our findings that cell fusion is equally inhibited in scramble and PKCθ^shRNA^ myotubes treated with a MEK1/2 inhibitor suggests that MEK signaling is required for fusion independent of PKCθ. Additionally, abrogation of PKCθ promoted full completion of the myogenic program and increased rates of protein synthesis, despite reduced IR phosphorylation and maintained higher protein synthesis rates when treated with a PI3-kinase inhibitor. These findings demonstrate that PKCθ may be a viable therapeutic target to promote increases in protein synthesis and promote the maintenance of skeletal muscle health in conditions with impaired insulin signaling.

## Methods

### C_2_C_12_ ShRNA infection

C_2_C_12_ mouse muscle cells were provided by Francis X. Pizza (University of Toledo, Dept. of Kinesiology). To identify an siRNA to knockdown mouse PKCθ (mPKCθ) a free Web-based tool (http://www.genelink.com/sirna/shRNAi.asp) was used to design a putative siRNA against the mPKCθ gene and to design oligonucleotides that encode a corresponding small hairpin RNA (shRNA) as previously described [51]. Origene was utilized to construct the shRNA plasmid with oligonucleotides: ACCGTTTCTTCGAATCGGTTTATCCAACT and the homologous sequence. The mPKCθ shRNA construct was co-transfected together with vectors expressing gag-pol, REV and VSV-G into 293FT cells (Invitrogen) to generate a third generation lentiviral construct. Transfection was achieved using Lipofectamine 2000 (Invitrogen, Carlsbad, CA) using 100 ng total DNA per cm^2^ of the growth plate or well. The supernatants were harvested and the cell debris was removed by centrifugation at 2000 g. After addition of polybrene (5 ng/ml, Sigma Chemical Co., St. Louis, MO), the supernatant was used to infect C_2_C_12_ cells to establish a cell line that has mPKCθ stably down regulated (PCKθ^shRNA^) and a scramble shRNA control. After 72 hours the cells were selected by puromycin.

### Cell culture

Scramble and PKCθ^shRNA^ cells were seeded in tissue culture treated 6 well plates at equal density. They were grown in Hyclone DMEM (Fisher Scientific) supplemented with antibiotics and heat inactivated Hyclone FBS (Fisher Scientific) at a final concentration of 10% (growth media). To promote myoblast differentiation and fusion, ~90% confluent cultures were serum deprived by switching to DMEM containing horse serum (Fisher Scientific) at a final concentration of 2% (differentiation media). The day that growth media was replaced with differentiation media is considered Day 0. Cells were maintained in differentiation media for 4 days and then processed for immunoflourescence or protein extraction. Media was changed every 48 hours except when indicated.

### PI3-kinase and MEK1/2 inhibition

Beginning on Day 0, scramble and PKCθ^shRNA^ cells were incubated in differentiation media supplemented with the PI3-kinase inhibitor wortmannin (Cayman Chemicalor the MEK1/2 inhibitor U0126 (Cayman Chemical) at a final concentration of 10 μM. Media was changed daily with fresh inhibitor. Following 4 days of treatment, cells were processed for immunoflourescence. To confirm inhibition of PI3-kinase and MEK1/2 with wortmannin and U0126 respectively, confluent myoblasts were serum starved overnight and treated with 10nM insulin in the presence or absence of wortmannin or U0126. Cells were analyzed for AKT serine 473 phosphorylation and ERK threonine 202/tyrosine 204 phosphorylation as an indicator of drug effectiveness as described below.

### Immunofluorescence

Following 4 days of differentiation, wells were washed with PBS and fixed with cold 70% methanol/30% acetone for 10 min at room temperature. Cells were permeabilized with 0.05% triton-x 100 and blocked for 30 min at room temperature. Wells were incubated with anti-sarcomeric myosin heavy chain (MHC) MF20 (developed by Donald A. Fischman and obtained from the Developmental Studies Hybridoma Bank, The University of Iowa, Department of Biology, Iowa City, IA 52242) diluted 1:20 in blocking buffer for 2 hours at room temperature. Wells were washed and incubated with goat anti-mouse FITC secondary antibody (Invitrogen) diluted 1:200 in PBS for 30 min at room temperature. Cover slips were mounted with Vector Sheild containing 4',6-diamidino-2-phenylindole (DAPI) (Vector Labs).

### Myoblast fusion

MHC positive (MHC+) cells were viewed at 10X magnification. To quantify cell fusion, 5 fields were viewed per well in a predetermined manner by a blinded investigator; starting from the center of the well, the stage was moved two complete fields to the right (field 1), two fields up (field 2), four fields to the left (field 3), two fields down (field 4), and 4 fields to the right (field 5). For each field, one picture of MHC+ cells and one picture of DAPI labeled nuclei were taken and merged. A blinded investigator chose 10 MHC+ cells per field. The total number of nuclei were counted in 50 MHC+ cells per well and repeated in 3 wells for PKCθ^shRNA^ and scramble cell lines. This yielded a total of 150 MHC+ cells analyzed for each cell line.

### Myotube density

Density quantification (degree of MHC+ cell coverage of each well) using ImagePro Plus software was performed on images taken to determine myoblast fusion. The average MHC+ density (arbitrary units) across all 5 images per well was determined in 3 independent wells per condition and cell line.

### Real time PCR

RNA was extracted using a commercially available kit according to the manufacturer’s instructions (5 Prime). Following quantification using a Nanodrop (Thermo Fisher), 1ug of total RNA was reverse transcribed using a high capacity cDNA synthesis kit (Applied Biosystems). Real time PCR was performed on a Applied Biosystems Step One Plus system (Applied Biosystems). Fold change in mRNA levels was determined using 2^-ΔΔ^ Ct with GAPDH as a control gene. Primer sequences used were as follows: GAPDH forward ATGTTTGTGATGGGTGTGAA, GAPDH reverse ATGCCAAAGTTGTCATGGAT, PKCθ forward TACATCCAGAAAAAGCCAACCA, PKCθ reverse TTCTGTCCGCCCATTGTTCT, PKCΔ forward GCCAGAAGTCTCTGGGAGTG, PKCΔ reverse AAAGCTGCCTTTGCCAAGTA, myogenin forward CGCGATCTCCGCTACAGA, myogenin reverse TGGGACCGAACTCCAGTG, PTK2 (focal adhesion kinase, FAK) forward GGTCCGACTGGAAACCAACA, FAK reverse GGCTGAAGCTTGACACCCTC, caveolin 3 forward ACAGCTTCGACGGTGTATGG, and caveolin 3 reverse GTGGAACACCCAGCAGTGTA.

### Western blot

Cells were collected in lysis buffer (Final concentration: 1X RIPA buffer (Millipore), 1% triton-x100, 3% SDS) supplemented with Halt Protease and phosphatase inhibitors (Pierce). Cells were lysed by continuous, vigorous shaking for 20 min at 4°C. Lysates were centrifuged and supernatants used to determine protein concentration by BCA (Pierce).

SDS-PAGE and transfer were performed as previously described [35]. Proteins were blocked for 1 hour at room temperature. Membranes were washed with TBS-tween 20 (0.1%) (TBST) and incubated overnight at 4°C with either rabbit anti-mouse PKCθ (C-18; Santa Cruz) diluted 1:100, MHC MF20 (Developmental Studies Hybridoma Bank), or one of the following from Cell Signaling Technologies diluted 1:500: rabbit anti-mouse PKCθ threonine 538, rabbit anti-mouse insulin receptor (IR) tyrosine 1345, mouse anti-mouse total IR, rabbit anti-mouse insulin receptor substrate 1 (IRS1) serine 1101, rabbit anit-mouse IRS1 serine 636/639, rabbit anti-mouse IRS1 tyrosine 1222, mouse anti total IRS1, rabbit anti-mouse AKT serine 473, rabbit anti-mouse threonine 308, mouse anti total AKT, rabbit anti-mouse mTOR serine 2448, mouse anti-mouse total mTOR, mouse anti-mouse ERK1/2 threonine202/tyrosine204, rabbit anti-mouse total ERK1/2, rabbit anti-mouse ERK5 threonine218/tyrosine 220, or rabbit anti-mouse total ERK5. Membranes probed for total PKCθ and MHC were normalized to mouse anti β-actin (Sigma Aldrich) diluted 1:15,000. After washing, the membrane was incubated for 2 hours at room temperature with species appropriate secondary antibodies (Licor) diluted 1:5000 in blocking buffer. Results were visualized using the Odyssey Imaging System (Licor). Band density was determined using Image J software and expressed as a fold change relative to scramble. Proteins probed for phosphorylation were normalized to respective total protein expression.

### Protein synthesis

Following 4 days in differentiation media in the presence or absence of wortmannin or U0126 replaced daily, cells were incubated for 1 hour in serum-free DMEM containing 2.5 mM phenylalanine and 2.5 *μ*Ci/ml [^3^H] phenylalanine (Perkin Elmer, Boston, MA, USA). After incubation, cells were washed with ice-cold PBS (pH 7.5) and proteins were precipitated with 10% trichloroacetic acid (TCA). An aliquot of the culture medium was saved to determine the specific activity of the medium (c.p.m). TCA homogenates were incubated on ice for 30 minutes; plates were thoroughly scraped followed by centrifugation at 4500 g for 5 min. The supernatant was discarded and the TCA insoluble fraction was resuspended in 10% TCA, followed by centrifugation at 4500 g for 5 min. This was repeated four consecutive times, and the TCA insoluble fraction was homogenized in 0.15 M NaOH at 55°C for 1 hour with frequent vortex mixing. Aliquots of each sample were analyzed to determine the incorporated radioactivity (c.p.m) via liquid scintillation counting using a Beckman Coulter LS 6500. Protein content was analyzed using the DC protein assay (Bio-Rad laboratories, Hercules, CA). The rate of protein synthesis was calculated as femtomoles [^3^H] phenylalanine per milligram of protein per hour.

### Statistical analysis

Statistical analyses were performed using Graph Pad Prism 5 software. Student’s t-Test was used to determine differences between two means. One-Way ANOVA was used to determine differences when more than 2 variables were compared, followed by a Bonferroni multiple comparisons post-hoc test. Analysis of gene expression between cell types across time was performed using a two-way ANOVA. Significance required an alpha level of p<0.05.

## Abbreviations

PKCθ: Protein kinase C theta; PKCΔ: Protein kinase C delta; IR: Insulin receptor; IRS1: Insulin receptor substrate-1; AKT: Also known as protein kinase B (PKB); ERK: Extracellular signal-related kinase; MEK1/2: Mitogen activated ERK kinase; PI3-kinase: Phosphoinositide 3-kinase; mTOR: Mammalian target of rapamycin; shRNA: Short hairpin RNA.

## Competing interests

The authors declare no competing interests.

## Authors’ contributions

JSM contributed to experimental design, growth and treatment of cells, protein and gene expression analysis, and wrote the manuscript. TDH developed the scramble and PKCθ knockdown cell lines and contributed to experimental design. RAH performed protein synthesis experiments. EO and JLO contributed by quantifying cell differentiation and cell fusion. AD performed *in vitro* cell staining. TJM, ERS and JWH contributed to experimental design, interpretation of data and manuscript revising. JWH gave final approval for publication. All authors read and approved the final manuscript.
